# Protective Effect of Gamma Aminobutyric Acid against Aggravation of Renal Injury Caused by High Salt Intake in Cisplatin-Induced Nephrotoxicity

**DOI:** 10.3390/ijms23010502

**Published:** 2022-01-03

**Authors:** Hyesook Lee, Seon Yeong Ji, Hyun Hwangbo, Min Yeong Kim, Da Hye Kim, Beom Su Park, Joung-Hyun Park, Bae-Jin Lee, Gi-Young Kim, You-Jin Jeon, Yung Hyun Choi

**Affiliations:** 1 Department of Biochemistry, College of Korean Medicine, Dong-Eui University, Busan 47227, Korea; 14769@deu.ac.kr (H.L.); 14602@deu.ac.kr (S.Y.J.); ilytoo365@deu.ac.kr (M.Y.K.); 14983@deu.ac.kr (B.S.P.); 2 Anti-Aging Research Center, Dong-Eui University, Busan 47340, Korea; 3 Korea Nanobiotechnology Center, Pusan National University, Busan 46241, Korea; hbhyun2003@naver.com; 4 Department of Molecular Biology, Pusan National University, Busan 46241, Korea; believe0402@pusan.ac.kr; 5 Ocean Fisheries & Biology Center, Marine Bioprocess Co., Ltd., Busan 46048, Korea; pdc327@hanmail.net (J.-H.P.); hansola82@hanmail.net (B.-J.L.); 6 Department of Marine Life Science, Jeju National University, Jeju 63243, Korea; immunkim@jejunu.ac.kr (G.-Y.K.); youjinj@jejunu.ac.kr (Y.-J.J.)

**Keywords:** cisplatin, gamma-aminobutyric acid, kidney, nephrotoxicity, salt

## Abstract

Gamma-aminobutyric acid (GABA) is one of the inhibitory neurotransmitters. Several studies have suggested that GABA supplements can reduce blood pressure and modulate the renal immune system in vitro and in vivo. In the present study, we investigated the effect of GABA-enriched salt as an alternative to traditional salt on aggravated renal injury by high salt intake in cisplatin-induced nephrotoxicity mice. High salt intake accelerated the increase of biomarkers, such as blood urea nitrogen and serum creatinine levels for renal injury in cisplatin-induced nephrotoxicity mice. However, oral administration of GABA-contained salt notably suppressed serum BUN and creatinine levels. The efficacy of GABA salt was superior to lacto GABA salt and postbiotics GABA salt. Furthermore, GABA-enriched salt markedly restored histological symptoms of nephrotoxicity including renal hypertrophy, tubular dilation, hemorrhage, and collagen deposition aggravated by salt over-loading in cisplatin-exposed mice. Among them, GABA salt showed a higher protective effect against cisplatin-induced renal histological changes than lacto GABA salt and postbiotics GABA salt. In addition, administration of high salt significantly enhanced expression levels of apoptosis and inflammatory mediators in cisplatin-induced nephrotoxicity mice, while GABA-enriched salt greatly down-regulated the expression of these mediators. Taken together, these results demonstrate the protective effect of GABA against damage caused by high salt intake in cisplatin-induced renal toxicity. Its mechanism may be due to the suppression of hematological and biochemical toxicity, apoptosis, and inflammation. In conclusion, although the protective efficacy of GABA salt on renal injury is different depending on the sterilization and filtration process after fermentation with *L*. *brevis* BJ20 and *L*. *plantarum* BJ21, our findings suggest that GABA-enriched salt has a beneficial effect against immoderate high salt intake-mediated kidney injury in patients with cisplatin-induced nephrotoxicity.

## 1. Introduction

It has been known for decades that salt plays a critical role in the pathogenesis of renal disease and cardiovascular diseases including hypertension [1]. According to the World Health Organization (WHO), the restriction of sodium intake to less than 2.3 g/day of sodium corresponding to 5.8 g of salt (or 100 mmol) is one of the most cost-effective measures to improve public health [2]. However, current estimates demonstrate the mean intake of salt in Asian countries is higher than the global mean [3]. Salt over-loading not only increases albuminuria in individuals without primary renal disease but also raises excretion of albumin and protein in patients with renal diseases [4]. Several epidemiological studies have revealed that higher sodium intake can accelerate the progression of renal injury and cause a poor prognosis of renal diseases [5,6]. In addition, dietary supplementation of high salt can aggravate proteinuria and glomerulosclerosis in animal models with renal damages [7,8]. Furthermore, salt-sensitive individuals present an abnormal kidney reaction to salt intake [9]. Moreover, kidney damage can cause the onset of salt-sensitive hypertension, which is accompanied by kidney inflammation and fibrosis, leading to end-stage renal disease [10]. In this respect, reducing salt intake is essential to stop or delay the progression of kidney disease [11].

Gamma-aminobutyric acid (GABA) is one of the inhibitory neurotransmitters known to be synthesized in the brain from glutamic acid [12]. GABA is marketed in the US as a dietary supplement, its safety is verified by accumulated evidence on clinical studies, adverse event information, and toxicology data [13]. Numerous studies reported the beneficial effect on reducing stress and enhancing sleep, and on several biological activities, including anti-hypertension, anti-diabetes, anti-cancer, antioxidant, anti-inflammation, anti-microbial, and anti-allergy [14,15]. Among its biological effects, it is noteworthy that GABA supplements can reduce blood pressure in experimental hypertensive rodents and modulate the immune system in in vitro renal cells [16,17,18,19,20]. More recently, Son et al. suggested that GABA-contained salt made by fermentation with *Lactobacillus brevis* BJ20 can reduce blood pressure and renal function deterioration in high fat diet-induced hypertensive mice [21]. In that study, the hypotensive effect of GABA salt intake has been suggested to be attributed to suppression of vascular smooth muscle cell proliferation and endothelial cell dysfunction. Thus, GABA-enriched salt could be an alternative to traditional salt. Although numerous studies have demonstrated that GABA or GABA-enriched food supplementation can attenuate the progression of renal disease or hypertension, the effect of GABA salt intake on acute nephrotoxicity remains unclear. Thus, the objective of this study was to investigate the effect of GABA salt on aggravation of renal injury caused by high salt intake in cisplatin-induced acute nephrotoxicity. Whether the efficacy of GABA salt was changed depending on sterilization and filtration process after fermentation with *L. brevis* BJ20 and *L. plantarum* BJ21 was also assessed.

## 2. Results

### 2.1. Effect of GABA on Body Weight Loss and Renal Hypertrophy Caused by High Salt Intake in Cisplatin-Induced Acute Nephrotoxicity

**Figure 1:**
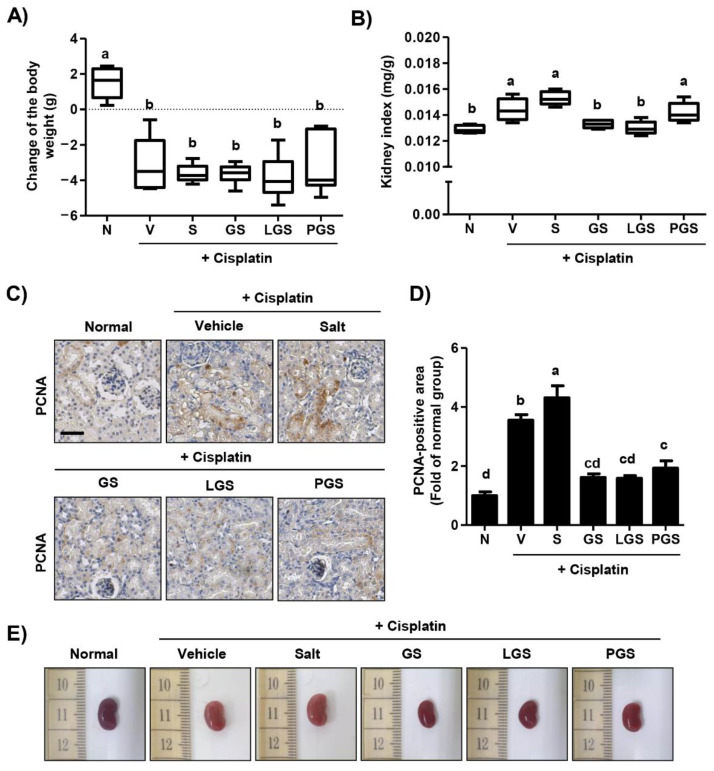
Effect of GABA on body weight loss and renal hypertrophy following high salt intake in cisplatin-induced acute nephrotoxicity. (**A**) Change of body weight. (**B**) Kidney/body weight ratio. Data are expressed as means ± SD (*n* = 8). ^a,b^ Bars with different letters are significantly different at *p* < 0.05 by Tukey test. (**C**) Intra-renal expression of proliferating cell nuclear antigen (PCNA). Scale bar = 100 μm. (**D**) Quantitative analysis for the positive area of PCNA. Data are expressed as means ± SD (*n* = 4). ^a–d^ Bars with different letters are significantly different at *p* < 0.05 by Tukey test. (**E**) Representative kidney images. N, normal group; V, cisplatin + saline group; S, cisplatin + 4% high salt group; GS, cisplatin + high salt containing gamma aminobutyric acid (GABA) group; LGS, cisplatin + 4% sodium containing lacto GABA salt group; PGS, cisplatin + 4% sodium containing postbiotics GABA salt group.

To examine the effect of GABA on body weight after high salt intake in cisplatin-induced acute nephrotoxicity mice, we calculated changes in body weight. As shown in Figure 1A, a single intraperitoneal injection of cisplatin caused a significant bodyweight loss. However, there were no significant differences in body weights of orally administered high salt groups with or without GABA. Although the relative organ weight of the kidney was markedly increased by cisplatin injection, such cisplatin-induced kidney hypertrophy was normalized by oral administration of GABA salt or probiotic GABA salt (Figure 1B). In contrast, postbiotic GABA salt treatment did not decrease the relative organ weight of the kidney. Next, to verify the protective effect of GABA salt against kidney hypertrophy induced by cisplatin and high salt intake, we performed immunohistochemistry to determine the expression of proliferating cell nuclear antigen (PCNA), a marker of cell proliferation, in renal tissues. Numerous PCNA positive-staining areas were seen in the renal tubule and cortex of the cisplatin vehicle group (Figure 1C,D). In addition, the over-expression of PCNA caused by cisplatin injection was greatly increased in the group administered with high salt. However, up-regulation of PCNA induced by cisplatin and high salt intake was substantially down-regulated in groups administered with GABA-enriched salt. These results indicated that GABA-enriched salt was involved in the normalization of kidney hypertrophy in cisplatin-induced nephrotoxicity, although it did not recover body weight loss. Meanwhile, it did not induce any alterations in relative organ weight of liver, heart, lung, spleen, or pancreas excluding kidney (data not shown). As shown in Figure 1E, the image of the kidney in the cisplatin-injected vehicle group was paler compared to the normal group. However, high salt-treated kidneys retained the color induced by cisplatin, while kidneys from GABA salt, lacto GABA salt and postbiotics GABA salt treated groups appeared redder.

### 2.2. Effect of GABA on Changes of Hematological and Biochemical Profiles following High Salt Intake in Cisplatin-Induced Acute Nephrotoxicity

Hematological analysis results showed that red blood cell (RBC) count, hematocrit, hemoglobin levels, mean corpuscular volume (MCV), mean corpuscular hemoglobin (MCH), and MCH concentration (MCHC) were not significantly different among groups (Table 1). Nevertheless, white blood cells (WBC) count and platelet count were significantly decreased after cisplatin injection, these levels were no statistically significant difference between the vehicle group and the high salt group, as well as GABA-contained salt groups. However, cisplatin-injected mice exhibited significant increases in serum BUN and creatinine levels than the normal group of mice. In addition, high salt intake markedly enhanced serum creatinine levels compared with the vehicle group. Oral administration of GABA-contained salt notably suppressed blood urea nitrogen (BUN) and serum creatinine levels, and the efficacy of GABA salt was superior to that of lacto GABA salt or postbiotics GABA salt. These results indicate that cisplatin can induce thrombopenia, leukopenia, and serum biochemical hallmarks of kidney damage and that high salt intake can partially aggravate cisplatin-induced hematological and biochemical changes. However, these alterations following either cisplatin or cisplatin and high salt combination were prominently improved by GABA salt treatment, whose effect was far better than that of lacto GABA salt or postbiotics GABA salt.

### 2.3. Effect of GABA on Renal Histologic Changes following High Salt Intake in Cisplatin-Induced Acute Nephrotoxicity

**Figure 2:**
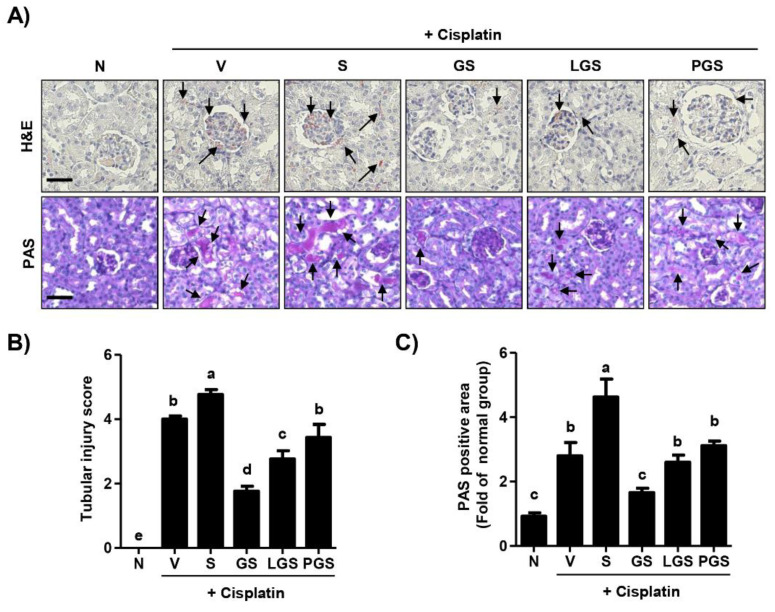
Effect of GABA on renal histological changes following a high salt intake in cisplatin-induced acute nephrotoxicity. (**A**, top) Representative images of H&E-stained sections in the kidney. Black arrows indicate histological changes including mononuclear cell infiltration, tubular dilation, and hemorrhage. Scale bar = 100 μm. (**A**, bottom) Representative images of periodic acid Schiff (PAS)-stained sections in the kidney. Black arrows indicate glycogen accumulation in injury renal tubules that stained with a strong pink color. Scale bar = 100 μm. (**B**) Tubular injury score evaluated on H&E-stained kidney sections. (**C**) Quantitative analysis for PAS-positive area. (**B**,**C**) Data are expressed as means ± SD (*n* = 3). ^a–e^ Bars with different letters are significantly different at *p* < 0.05 by Tukey test. N, normal group; V, cisplatin + saline group; S, cisplatin+ 4% high salt group; GS, cisplatin + high salt containing GABA group; LGS, cisplatin + 4% sodium containing lacto GABA salt group; PGS, cisplatin + 4% sodium containing postbiotics GABA salt group.

Histological changes of the kidney included mononuclear cell infiltration, tubular dilation, and hemorrhage in the cisplatin vehicle group (Figure 2A, top). Furthermore, oral administration of high salt gradually enhanced these histological changes caused by cisplatin. However, in the group treated with GABA salt, inflammatory infiltrated cells and hemorrhage were not noticed in the kidney. In addition, tubular dilation recovered to be histologically normal. Cisplatin-induced histological changes were also attenuated in the group treated with lacto GABA salt or postbiotic GABA salt, although their efficacy was less than that of GABA salt. Based on the result from H&E staining, Figure 2B showed that tubular injury score significantly enhanced in high salt uptake after cisplatin exposure, while this increased score significantly decreased in GS and LGS group. As shown in Figure 2A bottom, numerous glycogens were deposited in renal tubules of the cisplatin vehicle group. In addition, renal tubular epithelial damages, such as swelling, shedding, and glycogen accumulation more distinctly appeared in the cisplatin combined with the high salt intake group. In contrast, cisplatin-mediated renal tubular epithelial damages were markedly attenuated in groups treated with GABA salt, lacto GABA salt, and postbiotics GABA salt. Among them, GABA salt had the highest protective effect against cisplatin-induced histological changes in renal tissues. The quantitative data for the PAS-stained area indicated that high salt intake accelerated the PAS-stained area increasing in cisplatin injected mice, whereas this up-regulation by high salt was significantly suppressed by GABA salt and lacto GABA salt administration (Figure 2C).

### 2.4. Effect of GABA Salt on Renal Cell Death in Cisplatin-Induced Acute Nephrotoxicity

**Figure 3:**
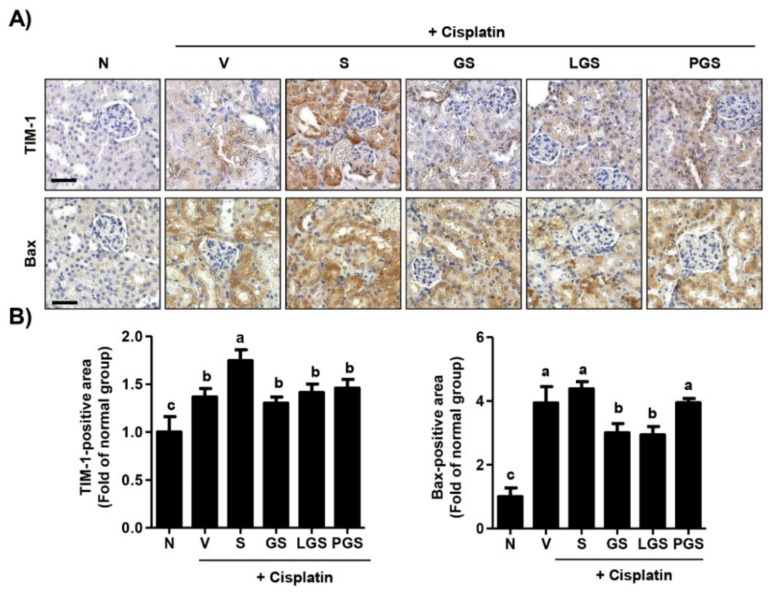
Effect of GABA salt on renal cell death in cisplatin-induced acute nephrotoxicity. (**A**) Intra-renal expression of T-cell immunoglobulin and mucin domain 1 (TIM-1) and Bcl2- associated X (Bax). Scale bar = 100 μm. (**B**) Quantitative analysis for positive area of TIM-1 and Bax. Data are expressed as means ± SD (*n* = 4). ^a–c^ Bars with different letters are significantly different at *p* < 0.05 by Tukey test. N, normal group; V, cisplatin + saline group; S, cisplatin+ 4% high salt group; GS, cisplatin + high salt containing GABA group; LGS, cisplatin + 4% sodium containing lacto GABA salt group; PGS, cisplatin + 4% sodium containing postbiotics GABA salt group.

To assess whether histological changes in the kidney induced by cisplatin and high salt administration were involved in renal cell death, we performed immunohistochemistry for the expression of renal tubular injury makers. As shown in Figure 3A,B, a large number of T-cell immunoglobulin and mucin domain 1 (TIM-1) positive-staining areas could be observed in the renal tubule and in the renal cortex of the cisplatin vehicle group. Additionally, overexpression of TIM-1 induced by cisplatin injection was substantially enhanced by high salt intake. However, such up-regulation of TIM-1 by cisplatin and high salt intake was markedly suppressed by GABA salt, lacto GABA salt, or postbiotics GABA salt. Likewise, the expression of pro-apoptotic factor Bcl2- associated X (Bax) was also significantly increased in groups treated with cisplatin and high salt, while this over-expression was markedly decreased by GABA salt and lacto GABA salt intake. These results showed that high salt intake aggravated the over-expression of renal cell death markers induced by cisplatin, whereas this event was greatly attenuated by oral administration of GABA-enriched salt.

### 2.5. Effect of GABA Salt on Renal Inflammation in Cisplatin-Induced Acute Nephrotoxicity

**Figure 4:**
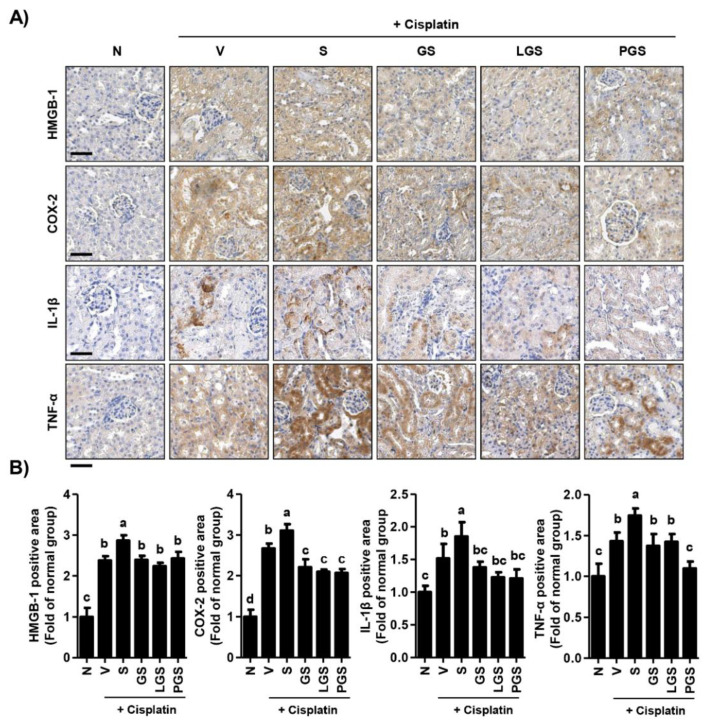
Effect of GABA salt on renal inflammation in cisplatin-induced acute nephrotoxicity. (**A**) Intra-renal expression levels of high mobility group box-1 (HMGB-1), cyclooxygenase-2 (COX-2), interleukin-1β (IL-1β), and tumor necrosis factor-α (TNF-α). Scale bar = 100 μm. (**B**) Quantitative analysis for the positive area of HMGB-1, COX-2, IL-1β and TNF-α. Data are expressed as means ± SD (*n* = 4). ^a–d^ Bars with different letters are significantly different at *p* < 0.05 by Tukey test. N, normal group; V, cisplatin + saline group; S, cisplatin+ 4% high salt group; GS, cisplatin + high salt containing GABA group; LGS, cisplatin + 4% sodium containing lacto GABA salt group; PGS, cisplatin + 4% sodium containing postbiotics GABA salt group.

In order to evaluate the effect of GABA salt on renal inflammation, we examined intra-renal protein expression levels of high mobility group box-1 (HMGB-1), cyclooxygenase-2 (COX-2), interleukin (IL)-1β, and tumor necrosis factor-α (TNF-α). HMGB-1, a potent trigger of inflammation, was strongly expressed in the renal cortex in cisplatin. Its expression levels were up-regulated by high salt intake (Figure 4A,B). However, cisplatin and high salt-induced over-expression of HMGB-1 was inhibited by oral administration of GABA salt, lacto GABA salt, or postbiotics GABA salt, although HMGB-1 expression levels were higher than those of the normal group. Furthermore, expression levels of pro-inflammatory mediators and cytokines including COX-2, IL-1β, and TNF-α were greatly increased in renal tubules of the group treated with cisplatin and high salt, while such over-expression was markedly decreased in groups treated with GABA-enriched salt. These results suggest that high salt intake can enhance the over-expression of renal inflammatory factors caused by cisplatin, whereas this event is suppressed by oral administration of GABA-enriched salt.

## 3. Discussion

Cisplatin is widely used as a potent chemotherapeutic drug because it is highly effective against various types of solid tumors, such as head and neck, lung, bladder, ovarian, cervical, breast, and gastric carcinomas [22,23]. However, it has been reported that cisplatin administration can cause several side effects, including ototoxicity, hepatotoxicity, gastrointestinal toxicity, and cardiotoxicity, with nephrotoxicity being the most common and notable clinical symptom [24,25]. Cisplatin accumulates in the proximal renal tubular epithelium and consequently provokes acute kidney injury. Nephrotoxicity has been estimated in 25 to 30% of patients undergoing cisplatin chemotherapy [22,26]. In this regard, the cisplatin-exposed rodent model has been recognized as a simple and reproducible model with high clinical relevance. It is widely accepted as a representative kidney disease model to investigate acute nephrotoxicity [27,28]. Numerous studies have verified that when an acute renal injury occurs, blood BUN, serum creatinine, and glomerular filtration rate are increased in cisplatin-exposed rodents [29,30,31]. Furthermore, it has been established that cisplatin can induce histological changes including mononuclear cell infiltration, hemorrhages, tubular dilation, and vacuolation in kidney tissues [30,32].

In the present study, we reproduced the cisplatin-induced acute nephrotoxicity model using C57BL/6 mice and verified that high salt intake aggravated symptoms of renal injury, whereas GABA salt had a protective effect against the synergic effect of cisplatin and high salt intake. Our results suggest that GABA salt supplementation can ameliorate increases of serum levels of biochemical parameters, such as BUN and creatinine levels induced by cisplatin and high salt intake (Table 1). Furthermore, GABA salt and lacto GABA salt intake can inhibit tubular dilation and hemorrhage aggravated by over-loading salt in a cisplatin-induced acute renal injury model (Figure 2). This histological result was supported by hematology findings showing that cisplatin decreased levels of WBC and platelet, while these levels were partially improved by GABA salt (Table 1). Moreover, our finding showed that high salt intake accelerated renal hypertrophy and the expression of proliferative marker PCNA in kidney tissues of cisplatin-exposed mice, whereas these negative alterations were suppressed by administration of GABA salt or lacto GABA salt (Figure 1). One special feature of renal injury is renal hypertrophy, an adaptive growth of kidney tissue to restore renal dysfunction, finally leading to tubular atrophy and interstitial fibrosis [33]. Therefore, commonly used endpoint criteria include renal fibrosis, histochemical staining for tubulointerstitial atrophy, fibrosis, and collagen deposition using PAS staining [34,35]. Our results from PAS staining showed that high salt intake accelerated renal fibrotic changes, such as swelling, shedding, and glycogen accumulation in cisplatin exposed mice, while these alterations were suppressed by GABA salt (Figure 2).

The primary pathophysiology of cisplatin-induced nephrotoxicity is characterized by proximal tubular injury and tubular epithelial cells apoptosis caused by inflammation, oxidative stress, or vasoconstriction [28,36]. In this context, the implication of apoptosis and inflammation in the pathogenesis of cisplatin-induced nephrotoxicity has been well established with accumulated evidence [37,38]. Faubel et al. [39] have demonstrated that cisplatin can up-regulate the expression of inflammatory cytokines and chemokines, such as IL-1β, IL-18, and TNF-α, resulting in inflammatory responses and leading to apoptosis and reduced glomerular filtration rate. Another study has reported that cisplatin can induce over-expression of pro-apoptosis and pro-inflammatory mediators, such as cleaved caspase-3, cleaved caspase-9, Bax, cyclooxygenase-2, and inducible nitric oxide synthase (iNOS) [31]. Furthermore, it has been suggested that cisplatin exposure can enhance the expression of pro-apoptotic mediators (including cleaved caspase-3 and -8) and decrease Bcl2/Bax ratio both in vitro and in vivo [38]. Park et al. [40] have reported that cisplatin can induce apoptosis in proximal tubule LLC-PK1 cells via activation of mitochondrial signaling pathways. In addition, it is well-known that high salt can enhance damage to the renal medullary cells, including apoptosis, DNA breaks, and mitochondrial dysfunction [41,42]. These previous findings were consistent with our present finding showing that injection of cisplatin could lead to over-expression of TIM-1, a sensitive marker for early detection of renal injury in animals with nephrotoxicity [43]. In the present study, our results showed that high salt intake enhanced the up-regulation of TIM-1 and Bax induced by cisplatin (Figure 3). Additionally, expression levels of pro-inflammatory mediators, such as HMGB-1, IL-1β, IL-6, and TNF-α were induced by high salt intake in cisplatin-induced nephrotoxicity mice (Figure 4). However, this over-expression induced by cisplatin and high salt intake was markedly decreased by GABA salt and lacto GABA salt intake. Notably, it has been established that GABA is an inhibitor of inflammation by decreasing pro-inflammatory mediator production and ameliorating inflammatory symptoms [44]. In addition, GABA has emerged as a promising compound that is able to regulate cancer due to induction of apoptosis and inhibition of proliferation and metastasis [44]. Results of the present study are partially consistent with previous studies demonstrating that GABA-enriched salt can restore renal function and reduce the production of inflammatory cytokines in high fat diet-mediated hypertensive mice [21]. Findings of the present study indicate that high salt intake can aggravate the over-expression of renal cell death mediators and pro-inflammatory mediators induced by cisplatin, whereas such effect could be greatly attenuated by oral administration of GABA-enriched salt.

Cisplatin-induced nephrotoxicity is a very complex disease, which involves both complex local events in the kidney and complex interconnected and interdependent systemic effects in the body [28]. Although the cisplatin rodent model is simple to induce and has many similarities with human cisplatin nephrotoxicity, there are also limitations that need to be addressed in further studies. It was argued that it lacks resemblance with human acute kidney injury in some aspects, such as renal morphological changes, and the impact of many interventions on the chemotherapeutic efficacy of cisplatin has not been adequately examined [28,45]. Furthermore, depending on the cisplatin dosage regimen rodents may develop not only different severity of nephrotoxicity but also extrarenal toxicity or even systemic toxicity [46]. In this respect, we considered that further studies are needed to identify the effect of GABA-enriched salts on the complexity of cisplatin’s characteristics and the influence of cisplatin dosage regiment on the result or study outcome. Furthermore, several studies suggested that renin angiotensin converting enzyme inhibitors and angiotensin II receptor blockers protect against cisplatin-induced nephrotoxicity, but no studies have yet been reported that cisplatin directly induces hypertension [47,48]. Therefore, we considered that further studies are required to investigate the effect of GABA salt on blood pressure and renin-angiotensin system in cisplatin-induced nephrotoxicity mice.

## 4. Materials and Methods

### 4.1. Preparation of GABA Salt, Lacto GABA Salt, and Postbiotics GABA Salt

GABA-enriched salt was prepared as previously described [21] with some modifications. Briefly, *L. brevis* BJ20 was inoculated into a seed medium (3% yeast extract, 1% glucose, 1% monosodium glutamate, and 95% water) previously sterilized at 121 °C for 15 min by autoclaving. Seed medium (10% *v*/*v*) was then inoculated into a sterilized main culture medium (2% yeast extract, 0.7% glucose, 32% L-glutamic acid, 50% rice germ hydrolysate solution, and 15.3% water) at 37 °C for 48 h followed by fermentation with *L. plantarum* BJ21 for 24 h. The fermented solution was further fermented with refined salt for 6 h. GABA salt, lacto GABA salt, and postbiotics GABA salt were prepared from the fermented salt solution by spray drying. GABA salt and postbiotics GABA salt were additionally processed through sterilization & filtration and sterilization, respectively, before spray drying. The refined salt with most impurities removed through filtration, precipitation, and concentration processes using a large amount of seawater was purchased from Hanju Corp. (Yangju, Korea). GABA salt, lacto GABA salt, and postbiotics GABA salt were composed of 79.9, 71.3, and 79.4 g/100 g sodium chloride with 92.69, 111.92, and 97.25 mg/g GABA, respectively.

### 4.2. Animals and Experimental Procedure

This study was approved by the Institutional Animal Care and Use Committee of Dong-eui University (approval No. R2021-013). All experiments were conducted according to the guide for the care and use of laboratory animals. Forty-eight C57BL/6J mice (6-week-old, male) were purchased from Koatech Co. Ltd. (Pyeongtaek, Korea). After acclimatization for a week, these mice were randomly divided into six groups: Normal group (N; saline, *n* = 8); vehicle group (V; cisplatin + saline, *n* = 8); high salt group (S; cisplatin + commercial salt containing 4% NaCl, *n* = 8); GABA salt group (GS; cisplatin + GABA salt containing 4% NaCl, *n* = 8); lacto GABA salt group (LGS; cisplatin + lacto GABA salt containing 4% NaCl, *n* = 8); and postbiotics GABA salt group (PGS; cisplatin + postbiotics GABA salt containing 4% NaCl, *n* = 8). Acute kidney injury was induced by intraperitoneal injection of 20 mg/kg body weight cisplatin (Sigma-Aldrich Chemical Co., St. Louis, MO, USA) for all groups except for the normal group. Cisplatin was dissolved in normal saline (JW Pharmaceutical, Seoul, Korea). Saline, salt, GABA salt, Lacto GABA salt, and postbiotic GABA salt were orally administered over three times: at 1 h before cisplatin injection, at 24 h after cisplatin injection, and at 48 h after cisplatin injection. At 24 h after the last oral administration, mice were euthanized under CO_2_ euthanasia. During the study period, all mice were given a normal chow diet available ad libitum. Blood was collected to assess hematological and biochemical changes. The kidney was weighed after isolation and trimming. Relative organ weight was then analyzed.

### 4.3. Hematological and Biochemical Analysis

Whole blood was collected directly from the heart, placed into heparinized tubes, and allowed to clot for 30 min at room temperature. Thereafter, the blood was centrifuged at 3000 rpm for 10 min at 4 °C to obtain serum, which was stored at −80 °C for measurement of blood urea nitrogen (BUN) and serum creatinine. Whole blood was analyzed using a Sysmex XN-9000 analyzer (Sysmex Corporation, Kobe, Japan). BUN and creatinine levels were subsequently measured using a Cobas 8000 C702 chemistry analyzer (Roche, Mannheim, Germany).

### 4.4. Histopathological Analysis

Surgically isolated kidneys were preserved in 10% neutral buffer formalin and processed for routine paraffin embedding. Tissues were cut into 5 μm-thickness sections. Sections were stained with hematoxylin and eosin (YD Diagnostics Co., Yongin, Korea), as previously described [49]. Histopathological changes were blindly scored by three independent observers as follows: 0, no damage; 1, <10% damage; 2, 10–25% damage; 3, 25–50% damage; 4, 50–75% damage; and 5, >75% damage [15]. Three randomly chosen high-power fields per mouse kidney were used for counting. Meanwhile, other sections were subjected to periodic acid-Schiff (PAS; Sigma-Aldrich Chemical Co.) staining according to the manufacturer’s protocol [50]. Stained slides were observed using an EVOS FL Auto 2 imaging system (Thermo Fisher Scientific, Waltham, MA, USA).

### 4.5. Immunohistochemical Examination

To investigate changes of markers involved in kidney damage and inflammation, immunohistochemical analysis was performed for kidney tissues. Briefly, kidney sections with a thickness of 5 μm were deparaffinized, hydrated, processed in antigen retrieval solution (Abcam Inc., Cambridge, UK), and exposed to 3% hydrogen peroxide solution (Sigma-Aldrich Chemical Co.). After blocking with bovine serum albumin (Sigma-Aldrich Chemical Co.), slides were incubated at 4 °C overnight with primary antibodies for TIM-1 (Thermo Fisher Scientific., Cat. no. PA5-98302, 1:50 dilution), PCNA (Santa Cruz Santa Biotechnology Inc., Santa Cruz, CA, USA; Cat. no. sc-56, 1:100 dilution), Bax (Santa Cruz Santa Biotechnology Inc., Cat. no. sc-7480, 1:100 dilution), HMGB-1 (Abcam Inc., Cat. no. ab79823, 1:50 dilution), COX-2 (Abcam Inc., Cat. no. ab15191, 1:100 dilution), IL-1β (Cell Signaling Technology, Beverly, MA, USA, Cat. No. 12242, 1:100 dilution), and TNF-α (Cell Signaling Technology, Cat. No. 3707, 1:100 dilution) as previously described [51]. Subsequently, slides were incubated with secondary antibodies (DAKO Corp, Glostrup, Denmark) at room temperature for 1 h. Color development was performed by incubating slides with diaminobenzidine chromogen. Slides were counterstained with Mayer’s hematoxylin [52]. These stained slides were photographed using a fluorescence microscope (Carl Zeiss, Oberkochens, Germany) at the Core Facility Centerfor Tissue Regeneration, Dong-eui University (Busan, Korea). Quantitative analysis of histological staining was performed using a “threshold tool” of ImageJ^®^ (National Institutes of Health, Bethesda, MD, USA).

### 4.6. Statistical Analysis

Data are presented as mean ± standard deviation. One-way analysis of variance and post-hoc analyses were performed for comparisons between groups using GraphPad Prism 5.03 (GraphPad Software Inc., La Jolla, CA, USA). Statistical significance was set at *p* < 0.05.

## 5. Conclusions

**Figure 5:**
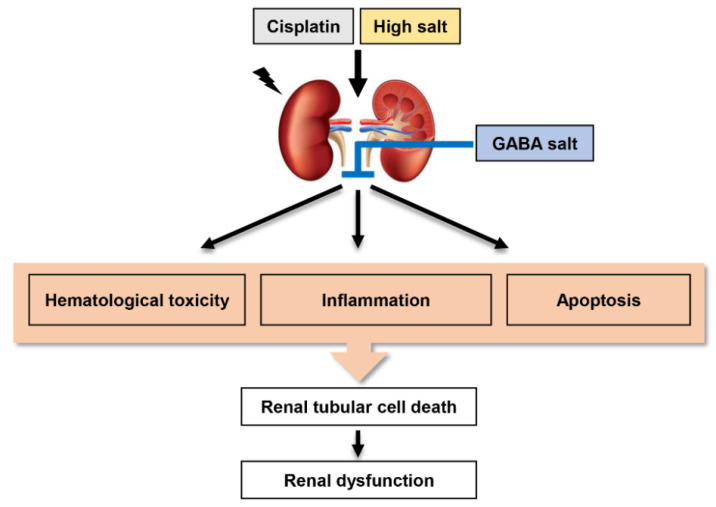
Schematic diagram of potential role of GABA-enriched salt in the negative effect of high salt intake on cisplatin-induced renal toxicity. GABA has a protective ability against negative effect of high salt intake in cisplatin-induced renal toxicity. Its mechanism involves suppression of hematological and biochemical toxicity, apoptosis, and inflammation.

GABA has a protective ability against the negative effect of high salt intake in cisplatin-induced renal toxicity. Its mechanism involves the suppression of hematological and biochemical toxicity, apoptosis, and inflammation (Figure 5). Although further studies are needed to confirm the renal protective effect of GABA in the renal cells and the clinical trials, our present findings suggest that GABA-enriched salt has a beneficial effect against immoderate high salt intake-mediated kidney injury in patients with cisplatin-induced nephrotoxicity. Among them, GABA salt has the best protective effect against cisplatin-induced histological changes in renal tissues. The protective effect of GABA salt on renal injury is different depending on the sterilization and filtration process after fermentation with *L. brevis* BJ20 and *L. plantarum* BJ21. Further studies are needed to identify its action mechanism depending on the process.

## Figures and Tables

**Table 1:** Changes of hematological and biochemical profiles after orally administered GABA-enriched salt in cisplatin-induced nephrotoxicity.

		+Cisplatin				
	Normal	Vehicle	Salt	GS	LGS	PGS
RBC (10^6^/μL)	10.14 ± 0.37 ^NS^	10.55 ± 0.56	10.71 ± 0.51	10.67 ± 0.39	10.86 ± 0.26	10.81 ± 0.56
WBC (10^3^/μL)	2.43 ± 0.69 ^a^	1.27 ± 0.31 ^b^	1.28 ± 0.32 ^b^	1.62 ± 0.23 ^b^	1.35 ± 0.12 ^b^	1.30 ± 0.33 ^b^
Hematocrit (%)	57.30 ± 2.51 ^NS^	57.15 ± 2.74	59.45 ± 2.33	58.77 ± 1.85	60.22 ± 2.05	60.62 ± 3.12
Hemoglobin (g/dL)	16.10 ± 0.71 ^NS^	16.43 ± 0.87	16.75 ± 0.87	16.78 ± 0.50	16.96 ± 0.67	17.07 ± 0.62
MCV (fL)	56.57 ± 0.99 ^NS^	55.23 ± 1.17	55.08 ± 0.53	55.91 ± 1.20	55.07 ± 0.86	56.16 ± 0.42
MCH (pg)	16.13 ± 0.40 ^NS^	15.87 ± 0.28	15.68 ± 0.10	15.71 ± 0.23	15.67 ± 0.28	15.70 ± 0.42
MCHC (g/dL)	28.09 ± 0.64 ^NS^	28.73 ± 0.27	28.51 ± 0.50	28.33 ± 0.63	28.30 ± 0.86	27.74 ± 0.84
Platelet (10^3^/μL)	922.75 ± 121.44 ^a^	599.83 ± 128.30 ^bc^	450.43 ± 132.07 ^c^	652.83 ± 104.90 ^b^	601.67 ± 82.69 ^bc^	596.00 ± 124.38 ^bc^
AST (U/L)	101.90 ± 15.30 ^NS^	93.55 ± 16.89	90.84 ± 20.84	96.01 ± 17.40	95.70 ± 31.31	103.96 ± 19.24
ALT (U/L)	19.55 ± 2.20 ^NS^	24.68 ± 3.17	22.15 ± 2.40	19.03 ± 6.89	20.47 ± 5.79	22.33 ± 6.03
ALP (U/L)	265.72 ± 37.43 ^NS^	236.38 ± 13.16	244.94 ± 26.36	223.56 ± 68.77	224.13 ± 52.65	223.08 ± 30.54
BUN (mg/dL)	14.31 ± 0.54 ^c^	18.74 ± 1.45 ^a^	20.46 ± 2.83 ^a^	16.15 ± 1.53 ^b^	18.86 ± 1.65 ^a^	19.21 ± 1.93 ^a^
Creatinine (mg/dL)	0.40 ± 0.06 ^d^	0.60 ± 0.06 ^bc^	0.69 ± 0.05 ^a^	0.56 ± 0.02 ^c^	0.62 ± 0.03 ^b^	0.66 ± 0.03 ^ab^

Data are expressed as means ± SD (*n* = 8). ^a–d^ Different letters over each column indicate significant differences between groups at *p* < 0.05 by Tukey test. ^NS^, no significance; RBC, red blood cells; WBC, white blood cells; MCV, mean corpuscular volume; MCH, mean corpuscular hemoglobin; MCHC, MCH concentration; AST, aspartate aminotransferase; ALT, alanine aminotransferase; ALP, alkaline phosphatase; BUN, blood urea nitrogen.

## Data Availability

Data presented in this study are available within the article. Other data that support findings of this study are available upon request from corresponding authors.
